# Blocking Interleukin-33 Alleviates the Joint Inflammation and Inhibits the Development of Collagen-Induced Arthritis in Mice

**DOI:** 10.1155/2020/4297354

**Published:** 2020-11-05

**Authors:** Yan Li, Yeqin Fu, Huan Chen, Xiaojin Liu, Mingcai Li

**Affiliations:** ^1^Department of Immunology, Ningbo University School of Medicine, Ningbo, China; ^2^Department of Clinical Laboratory, The Fifth Hospital of Shijiazhuang, Shijiazhuang, China

## Abstract

Rheumatoid arthritis (RA) is considered a systemic chronic inflammatory joint disease characterized by chronic synovitis and cartilage and bone destruction. Interleukin-33 (IL-33) is a proinflammatory cytokine which is highly expressed in the synovium of RA patients and the joints of mice with collagen-induced arthritis (CIA) and exacerbates CIA in mice. However, the role of the IL-33-neutralizing antibody in the murine model of CIA remains unclear. In the present study, CIA mice were given intraperitoneally with polyclonal rabbit anti-murine IL-33 antibody (anti-IL-33) or normal rabbit IgG control after the first signs of arthritis. Administration of anti-IL-33 after the onset of disease significantly reduced the severity of CIA and joint damage compared with controls treated with normal rabbit IgG. Anti-IL-33 treatment also significantly decreased the serum levels of interferon-*γ*(IFN-*γ*),IL-6, IL-12, IL-33, and tumor necrosis factor-*α* (TNF-*α*). Moreover, anti-IL-33 treatment significantly downregulated the production of IFN-*γ*, IL-6, IL-12, IL-33, and TNF-*α* in ex vivo-stimulated spleen cells. Together, our results indicate that the IL-33-neutralizing antibody may provide a therapeutic strategy for RA by inhibiting the release of proinflammatory cytokines.

## 1. Introduction

Interleukin-33 (IL-33) is a new member of the IL-1 family, which plays a biological role by binding the orphan receptor suppression of tumorigenicity 2 (ST2) (also called ST2L, IL-33R*α*, DER4, Fit-1, T1, IL-1RL1, or IL-1R4) [1]. Upon binding to IL-33, the ST2 receptor together with the IL-1 receptor accessory protein (IL-1RAcP) forms a functional signaling heterodimeric complex [1]. There are two forms of ST2, the soluble ST2 (sST2) and the membrane-bound ST2 (ST2L). The sST2 functions as a decoy receptor that can block IL-33/ST2 interaction [2]. ST2 is specifically expressed on mast cells and T helper 2 (Th2) cells and has been proved to be an important effector molecule of Th2 responses in murine collagen-induced arthritis (CIA) [3, 4]. IL-33 can activate nuclear factor-*κ*B (NF-*κ*B) and mitogen-activated protein kinases (MAPK) through binding with ST2 receptor and induce Th2 cytokine expression, leading to serious pathological changes in mucosal tissues [1]. IL-33 has many effects on inflammatory cells. It can induce mast cells and Th2 cells to produce proinflammatory and Th2 cytokines [1, 5–11], induce Th2 cell chemotaxis [12] and neutrophil migration [13], promote eosinophil and basophil adhesion, and enhance eosinophil survival and basophil migration [14–16]. IL-33 is involved in the pathogenesis of rheumatoid arthritis (RA) [17].

IL-33 levels are increased in sera and synovial fluid of patients with RA [18–20]. Increased IL-33 in sera and synovial fluid is associated with disease activity [21, 22] and bone erosion [23, 24] in RA. Elevated levels of sST2 are associated with RA disease activity and ameliorated inflammation in synovial fibroblasts [25]. In animal studies, IL-33 exacerbates antigen-induced arthritis (AIA) and autoantibody-induced arthritis by activating mast cells [26, 27]. In addition, Palmer et al. [4] demonstrated blockade of IL-33 signaling using an anti-ST2 antibody attenuates the severity of experimental arthritis. However, several studies showed that the development of AIA and CIA, as assessed by clinical or histological evaluation, is not impaired in IL-33-deficient mice [28], and administration of IL-33 or an IL-33 receptor (ST2L) antibody inhibited cartilage destruction, systemic bone loss, and osteoclast differentiation [29]. These studies indicate that the effect of IL-33 on experimental arthritis seems to be contradictory. The role of IL-33 in the pathogenesis of RA needs further study.

We have previously reported that polyclonal rabbit anti-murine IL-33 antibody (anti-IL-33) reduced cigarette smoke-induced lung inflammation [30] and allergic airway inflammation [31] in mice. In the present study, we provide novel evidence that anti-IL-33 suppresses the expression of interferon-*γ* (IFN-*γ*), IL-6, IL-12, IL-33, and tumor necrosis factor-*α* (TNF-*α*) and the subsequent joint destruction in collagen-induced arthritic mice. Our data suggest that the treatment of arthritis with the IL-33-neutralizing antibody is a promising new method, which may help to prevent joint damage.

## 2. Materials and Methods

### 2.1. Animals

Male DBA/1 mice (8–10 weeks old and weighting 20–24 g) were purchased from Shanghai SLAC Laboratory Animal Co. Ltd. (Shanghai, China). The mice were housed in pathogen-free conditions at the Animal Center of Ningbo University. Water and food were provided ad libitum. All experiments were approved by the Animal Ethics Committee of Ningbo University. The anti-IL-33 antibody and normal rabbit IgG were prepared as previously described [31, 32].

### 2.2. Induction of CIA and Administration of Anti-IL-33

**Figure 1:**
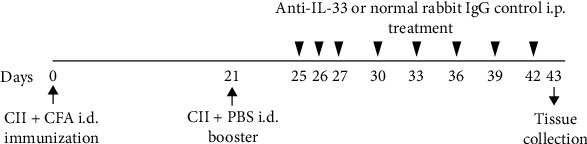
Induction of experimental arthritis and anti-IL-33 treatment. DBA/1 mice were immunized by an intradermal (i.d.) injection of bovine type II collagen (CII) emulsified in complete Freund's adjuvant (CFA) on day 0 and an intraperitoneal (i.p.) booster injection of CII dissolved in phosphate-buffered saline (PBS) on day 21. To evaluate the effect of anti-IL-33 on experimental arthritis, mice (*n* = 10) were injected i.p. with anti-mouse IL-33 (150 *μ*g/mouse) on days 25, 26, 27, 30, 33, 36, 39, and 42. The control group was injected with the same dose of control IgG (*n* = 10). On day 43, mice were killed, blood was collected, and joints were used for histological analysis.

CIA was elicited in DBA/1 mice as previously described with a slight modification [4]. Briefly, bovine type II collagen (CII; Chondrex) was diluted in 0.05 M acetic acid to a concentration of 2 mg/ml. It was emulsified in an equal amount of complete Freund's adjuvant (CFA) (5 mg/ml *Mycobacterium tuberculosis*, strain H37Ra; Difco, Detroit, MI). Mice were immunized intradermally (i.d.) at the base of the tail with 100 *μ*g of bovine CII. On day 21, mice were intraperitoneally (i.p.) injected with 100 *μ*g of CII dissolved in phosphate-buffered saline (PBS), and arthritis usually occurred a few days after the booster injection (Figure 1).

To study the effect of anti-IL-33 in murine CIA, DBA/1 mice (*n* = 10) were injected i.p. with anti-IL-33 from days 25 to 27 daily at a dose of 150 *μ*g/mouse and then once every 3 days until day 42. Control mice (*n* = 10) were injected with the same amount of normal rabbit IgG (Figure 1). The dose of 150 *μ*g anti-IL-33/mouse was based on previous studies by us [31] and Palmer et al. [4]. Mice were sacrificed by cervical dislocation at day 43 after immunization, and serum samples were obtained by aortic puncture. The joints and spleens were collected for histological examination and in vitro studies.

### 2.3. Assessment of Arthritis

Mice were monitored for signs of arthritis as previously described [3]. From day 20 after the first immunization onward, mice were examined daily for the onset of clinical arthritis. Mice were thought to have arthritis when significant redness and/or swelling were found in the digits or in other parts of the paws. Scores were assigned based on erythema, swelling or loss of function present in each paw on a scale of 0-3, giving a maximum score of 12 per mouse [3]. Paw thickness was measured with a dial caliper.

### 2.4. Collagen-Specific In Vitro Culture

Spleens were removed on day 43 after primary immunization. Single-cell suspensions were prepared and cultured at 2 × 10^6^ cells/ml in RPMI 1640 medium containing 10% fetal calf serum at 37°C in 5% CO_2_. Cells were cultured with 100 *μ*g/ml of CII in 96-well plates. After 72 or 96 h, the supernatant was collected and stored at –80°C until determined for cytokine concentration.

### 2.5. Histologic Analysis

For histological assessment, mice were sacrificed and whole knee and/or ankle joints were removed and fixed with 10% neutral-buffered formalin. The specimens were decalcified with 5% formic acid and embedded in paraffin [33]. Tissue sections (5 *μ*m) were stained with hematoxylin and eosin (HE) or toluidine blue (Sigma-Aldrich). Arthritis was quantified by a “treatment-blind” observer, and each joint was scored according to the degree of inflammation, synovial hyperplasia, and corrosion as described previously [3, 34].

### 2.6. Determination of Cytokines and Anticollagen Antibody Levels

All cytokines and anticollagen antibody levels were detected by ELISA. IFN-*γ*, IL-6, IL-10, IL-12, IL-33, sST2, and TNF-*α* in serum or culture supernatants were determined with ELISA kits (Biosource, Camarillo, CA) according to the manufacturer's instructions. The titers of serum anti-collagen II antibody were determined according to the method previously reported [3].

### 2.7. Statistical Analysis

Data were expressed as mean ± SEM. Statistical significance was assessed by the two-tailed unpaired Student *t*-test or two-way ANOVA with Bonferroni's multiple comparison test, as indicated in the figure legends. Statistical analyses were performed using GraphPad Prism 7 (GraphPad Software Inc., San Diego, USA). Values of *p* < 0.05 were considered significant.

## 3. Results

### 3.1. Anti-IL-33 Treatment Inhibited Disease Progress of Murine CIA

**Figure 2:**
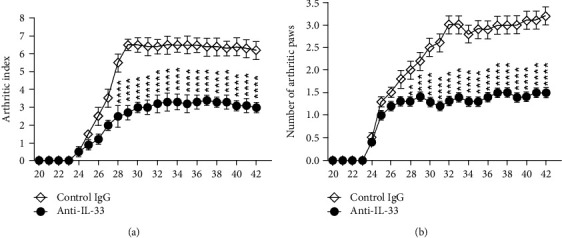
Anti-IL-33 inhibited the development of CIA in mice. From days 25 to 27, CIA mice were injected i.p. with anti-IL-33 or normal rabbit IgG (both 150 *μ*g/dose, *n* = 10) and then injected once every 3 days until day 42 for a total of 8 doses. According to arthritic index (a) and number of arthritic paws (b), the disease progression of mice was analyzed. Compared with the control IgG, anti-IL-33 treatment can significantly reduce the symptoms of arthritis in mice. Data are mean ± SEM (*n* = 10); ^∗∗^*p* < 0.01, ^∗∗∗^*p* < 0.001, and ^∗∗∗∗^*p* < 0.0001 vs. the control IgG group by two-way ANOVA with Bonferroni's multiple comparison test.

On day 24 after immunization, mice began to show clinical symptoms of arthritis. From days 25 to 27, mice were injected i.p. daily with anti-IL-33 or normal rabbit IgG, and then once every 3 days until day 42, for a total of 8 doses (Figure 1). The results showed that mice injected with control rabbit IgG reached the peak of arthritis on day 29. In contrast, mice treated with anti-IL-33 significantly attenuated disease in the arthritic index (Figure 2(a)) and number of arthritic paw (Figure 2(b)).

### 3.2. Anti-IL-33 Treatment Reduced Synovitis and Bone Erosion

**Figure 3:**
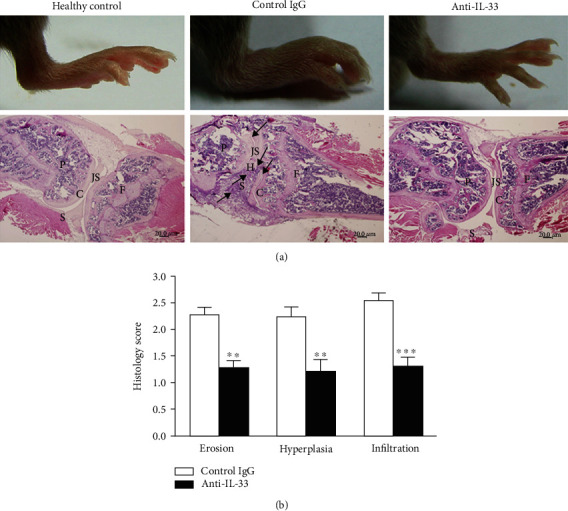
Anti-IL-33 treatment of mice with CIA resulted in reduced joint pathology. (a) On day 43, mice were killed and knee joints were collected and stained with HE. The inflammatory infiltrate, cartilage destruction, and bone erosion were observed in the control IgG-treated mice (arrows). P: patella; S: synovium; H: hyperplasia; JS: joint space; C: cartilage; F: femur. Magnification: ×40. (b) Anti-IL-33 antibody treatment alleviated the joint damage. The histological features of synovial bone erosion, hyperplasia, and cell infiltration were evaluated. Data are mean ± SEM (*n* = 8); ^∗∗^*p* < 0.01, ^∗∗∗^*p* < 0.001 vs. the control IgG group by two-way ANOVA with Bonferroni's multiple comparison test.

To examine whether anti-IL-33 administration reduced joint damage, histological evaluation of the cartilage and bone was performed. In the control group, there were obvious inflammatory cell infiltration, synovial hyperplasia, and adjacent cartilage and bone erosion (Figure 3(a)). However, anti-IL-33 treatment can significantly inhibit these pathological changes. The histological scores were shown in Figure 3(b). These data clearly indicate that anti-IL-33 can suppress the development of CIA and articular destruction.

### 3.3. Anti-IL-33 Treatment Suppressed Serum Levels of Proinflammatory Cytokines

**Figure 4:**
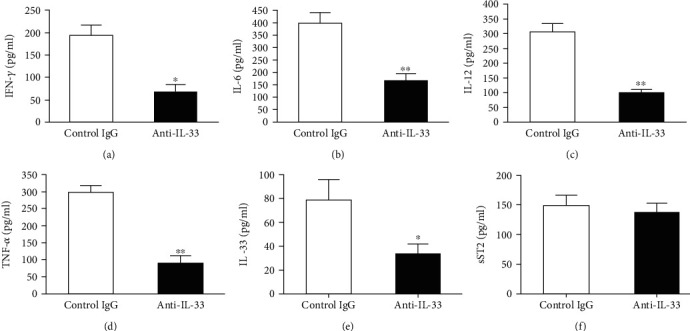
Effect of anti-IL-33 on serum proinflammatory cytokine production. Mice treated with anti-IL-33 or control IgG were killed on day 43, and the serum was collected from eight mice in each group. Levels of IFN-*γ* (a), IL-6 (b), IL-12 (c), TNF-*α* (d), IL-33 (e), and sST2 (f) were determined by ELISA. Data are mean ± SEM (*n* = 8); ^∗^*p* < 0.05, ^∗∗^*p* < 0.01 vs. the control IgG group by two-tailed unpaired Student's *t*-test.

To investigate the mechanism of action during IL-33 neutralization, serum levels of inflammatory cytokines were measured by ELISA. Compared with the control group, anti-IL-33 treatment significantly reduced serum IFN-*γ*, IL-6, IL-12, IL-33, and TNF-*α* levels, but sST2 levels did not change significantly (Figure 4). In addition, serum CII-specific IgG1 and IgG2a levels were similar in the two groups (data not shown).

### 3.4. Anti-IL-33 Treatment Inhibited CII-Specific Proinflammatory Immune Response In Vitro

**Figure 5:**
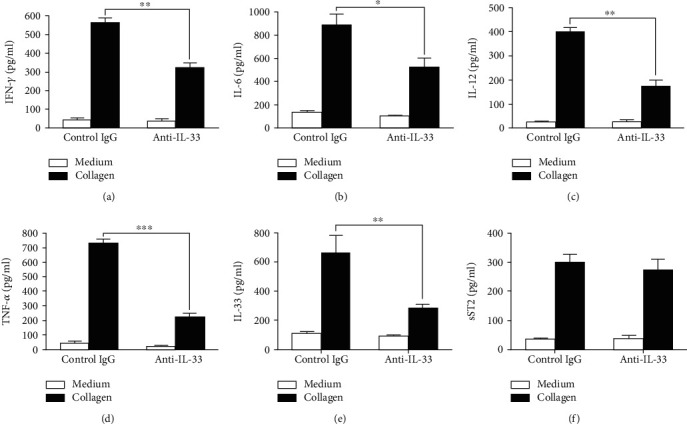
Anti-IL-33 decreases CII-specific proinflammatory cytokine production. Splenocytes (*n* = 5) were prepared from mice on day 43 and cultured with CII for 96 h. The content of cytokines in supernatants (72 h for IL-6, IL-12, IL-33, sST2, and TNF-*α*; 96 h for IFN-*γ* and IL-10) was detected by ELISA. Compared with the control group, the production of IFN-*γ* (a), IL-6 (b), IL-12 (c), TNF-*α* (d), and IL-33 (e) in spleen cell cultures of mice with anti-IL-33 treatment was significantly inhibited. There was no significant difference in the content of sST2 in spleen cells between the two groups (f). Data are mean ± SEM of triplicate cultures; *n* = 5. ^∗^*p* < 0.05, ^∗∗^*p* < 0.01, and ^∗∗∗^*p* < 0.001 by two-way ANOVA with Bonferroni's multiple comparison test.

We next examined the CII-specific inflammatory cytokine concentration in spleen cells. Compared with the control groups, the production of IFN-*γ*, IL-6, IL-12, IL-33, and TNF-*α* in spleen cells obtained at day 43 from anti-IL-33-treated mice was significantly reduced, while sST2 was not significantly changed (Figure 5). Additionally, the concentration of IL-10 in splenocytes of the two groups was similar (data not shown).

## 4. Discussion

In this study, we demonstrated that anti-IL-33 can reduce the severity of arthritis and the signs of joint injury in CIA mice after the onset of arthritis. Moreover, anti-IL-33 treatment was related to a significant suppression in proinflammatory cytokine (IFN-*γ*, IL-6, IL-12, IL-33, and TNF-*α*) production. These findings suggest that the IL-33-neutralizing antibody has a therapeutic effect on the onset of arthritis by inhibiting the activation of inflammatory cells and the production of proinflammatory cytokines.

IL-33 is considered to be an inflammatory cytokine in human RA and some experimental settings including mouse CIA. IL-33 was highly expressed in human RA synovium [35]. The levels of IL-33 were increased in fibroblast-like synoviocytes of RA patients [23]. The level of serum IL-33 was significantly correlated with the number of tender joints, C-reactive protein, disease activity score 28 joints, and leukocyte count and negatively correlated with the level of red blood cell and hemoglobin. At 3 and 6 months after treatment with etanercept (a human TNF antagonist), the average serum IL-33 level in RA patients had decreased significantly [36]. These findings suggest that IL-33 is involved in RA pathogenesis. In animal experiments, IL-33 mRNA was detected in the joints of CIA mice and increased in the early stage of the disease [4]. Moreover, IL-33 enhances autoantibody-mediated arthritis by promoting mast cell degranulation and proinflammatory cytokine production [27]. In contrast, the administration of antibodies that block ST2 signaling attenuated the severity of CIA [4]. Similarly, sST2, the decoy receptor for IL-33, significantly reduced the serious cellular infiltration, synovial hyperplasia, and joint erosion in the joints of mice [3]. The mechanism proposed to explain this effect was a direct inhibition of macrophage activation by sST2 via a putative sST2 receptor expressed at the macrophage surface [3]. However, other studies have shown that IL-33 appears to have an opposite effect on experimental arthritis. Palmer of University of Geneva group [28, 37] found that arthritis development is not impaired in absence of endogenous IL-33 in AIA, CIA, and K/BxN serum transfer-induced model of arthritis. Furthermore, Biton et al. [38] recently reported that IL-33 can inhibit the development of experimental arthritis by promoting the expansion of activated Foxp3^+^ regulatory T cells and establishing a type 2 immune response. They found that repeated injections of IL-33 during induction (early) and during development (late) of CIA strongly suppressed clinical and histological signs of arthritis. In contrast, a late IL-33 injection had no effect. These contradictory findings have been associated with environmental factors such as the different microbial colonization of experimental animals in different animal facilities [2]. In addition, a detailed understanding of interactions between ST2 with other signaling pathways seems to be mandatory for rational therapeutic manipulation of this system. These findings indicate that targeting IL-33 or its receptor might lead to radically different outcomes in patients (with arthritis). To the best of our knowledge, there is no report on the treatment of CIA or RA with the IL-33-neutralizing antibody. In the present study, we found that anti-IL-33 treatment can reduce the production of proinflammatory cytokines, thereby inhibiting the disease progress of murine CIA. Anti-IL-33 treatment significantly attenuated disease in the arthritic index and number of arthritic paw in CIA mice. Moreover, anti-IL-33 treatment reduced joint damage, including histological evaluation of the cartilage and bone integrity, infiltration of granulocytes and monocytes into the joint cavity, synovial hyperplasia, and cartilage and bone erosion.

In recent years, there is evidence that IL-33 is involved in the pathogenesis of RA by increasing the production of inflammatory molecules such as proinflammatory cytokines. A number of inflammatory mediators are involved in the inflammatory processes of RA. Some proinflammatory cytokines, including IL-1, IL-6, IL-8, IL-15, and TNF-*α*, are considered to be key molecules in the formation of RA inflammation.

Hong et al. [35] found that there is a significant positive correlation between IL-33 and IL-6 levels in the RA sera. Palmer et al. [4] reported that an anti-ST2 blocking antibody inhibits IL-33-induced IL-6 secretion. Xu et al. [26] showed that IL-33 increases the levels of IL-6 in vitro by bone marrow-derived mast cells from wild-type DBA/1 mice. Moreover, increased IL-33 leads to the upregulation of inflammatory cytokines such as IL-6 in patients with RA. In CIA mice, IL-33 induced the expression of proinflammatory cytokines such as IL-1*β*, IL-6, IL-13, granulocyte-macrophage colony-stimulating factor, and chemokines by bone marrow-derived mast cells from DBA/1 mice [26]. TNF-*α* is widely considered an important target of antirheumatic therapy. Blocking the TNF-*α* activity has been widely used to improve the progress of RA [39]. TNF-*α* significantly induced IL-33 mRNA expression and protein synthesis, and overexpression of IL-33 significantly increased TNF-*α*-induced IL-6, IL-8, and matrix metalloprotease (MMP)-3 in rheumatoid arthritis synovial fibroblast. On the contrary, IL-33 gene silencing significantly reduced the expression of IL-33, IL-6, IL-8, and monocyte chemotactic protein-1 (MCP-1) in TNF-*α*-induced RA synovial fibroblast [39]. Furthermore, sST2 reduced the production of IFN-*γ*, IL-17, TNF-*α*, and IL-10 in draining lymph nodes and decreased the levels of TNF-*α* and MMP-9 in the ankle joints of CIA mice [4]. IL-33 overexpression significantly increased TNF-*α*-induced expression of IL-6, IL-8, MCP-1, and MMP-3, while IL-33 gene silencing significantly decreased TNF-*α*-induced production of IL-6, IL-8, and MCP-1 [40]. The sST2 treatment also downregulated serum levels of IL-6, IL-12, and TNF-*α*. Spleen cells from the sST2-treated mice produced significantly less IFN-*γ*, TNF-*α*, IL-6, and IL-12 compared with cells from the control mice when cultured with collagen in vitro [3]. In this study, we treated CIA mice with the IL-33-neutralizing antibody and obtained similar results of sST2 treatment in CIA mice [3]. We detected that the production of IFN-*γ*, IL-6, IL-12, IL-33, and TNF-*α* in spleen cells of CIA mice treated with anti-IL-33 was significantly lower than that of control mice. Compared with the control group, anti-IL-33 treatment also significantly reduced the levels of serum IFN-*γ*, IL-6, IL-12, IL-33, and TNF-*α*. However, our data demonstrated that the neutralizing antibody against IL-33 had no significant effect on the production of sST2 in serum and spleen cells of CIA mice, suggesting that the inhibitory effect of anti-IL-33 may be independent of sST2. Altogether, our results showed that anti-IL-33 can inhibit the development of CIA in DBA/1 mice by downregulating the expression of proinflammatory cytokines IFN-*γ*, IL-6, IL-12, IL-33, and TNF-*α*.

In conclusion, our findings suggest that IL-33 may be a potential therapeutic target for RA. The IL-33-neutralizing antibody can decrease the joint inflammation and inhibit the development of CIA in mice. However, it is necessary to further investigate the role of the IL-33-neutralizing antibody in human RA. The treatment of the IL-33-neutralizing antibody may provide a new therapeutic approach for human RA.

## Data Availability

The data used to support the findings of this study are available from the corresponding author upon request.
